# Enabling Wearable Pulse Transit Time-Based Blood Pressure Estimation for Medically Underserved Areas and Health Equity: Comprehensive Evaluation Study

**DOI:** 10.2196/27466

**Published:** 2021-08-02

**Authors:** Venu Ganti, Andrew M Carek, Hewon Jung, Adith V Srivatsa, Deborah Cherry, Levather Neicey Johnson, Omer T Inan

**Affiliations:** 1 School of Electrical and Computer Engineering Georgia Institute of Technology Atlanta, GA United States; 2 The Wallace H. Coulter Department of Biomedical Engineering Georgia Institute of Technology Atlanta, GA United States; 3 VSNS, Inc Atlanta, GA United States

**Keywords:** wearable sensing, pulse transit time, cuffless blood pressure, noninvasive blood pressure estimation, health equity, mobile phone

## Abstract

**Background:**

Noninvasive and cuffless approaches to monitor blood pressure (BP), in light of their convenience and accuracy, have paved the way toward remote screening and management of hypertension. However, existing noninvasive methodologies, which operate on mechanical, electrical, and optical sensing modalities, have not been thoroughly evaluated in demographically and racially diverse populations. Thus, the potential accuracy of these technologies in populations where they could have the greatest impact has not been sufficiently addressed. This presents challenges in clinical translation due to concerns about perpetuating existing health disparities.

**Objective:**

In this paper, we aim to present findings on the feasibility of a cuffless, wrist-worn, pulse transit time (PTT)–based device for monitoring BP in a diverse population.

**Methods:**

We recruited a diverse population through a collaborative effort with a nonprofit organization working with medically underserved areas in Georgia. We used our custom, multimodal, wrist-worn device to measure the PTT through seismocardiography, as the proximal timing reference, and photoplethysmography, as the distal timing reference. In addition, we created a novel data-driven beat-selection algorithm to reduce noise and improve the robustness of the method. We compared the wearable PTT measurements with those from a finger-cuff continuous BP device over the course of several perturbations used to modulate BP.

**Results:**

Our PTT-based wrist-worn device accurately monitored diastolic blood pressure (DBP) and mean arterial pressure (MAP) in a diverse population (N=44 participants) with a mean absolute difference of 2.90 mm Hg and 3.39 mm Hg for DBP and MAP, respectively, after calibration. Meanwhile, the mean absolute difference of our systolic BP estimation was 5.36 mm Hg, a grade B classification based on the Institute for Electronics and Electrical Engineers standard. We have further demonstrated the ability of our device to capture the commonly observed demographic differences in underlying arterial stiffness.

**Conclusions:**

Accurate DBP and MAP estimation, along with grade B systolic BP estimation, using a convenient wearable device can empower users and facilitate remote BP monitoring in medically underserved areas, thus providing widespread hypertension screening and management for health equity.

## Introduction

### Background

Current clinical practice regarding hypertension management and control hinges on the century-old approach of obtaining infrequent cuff-based measurements of blood pressure (BP) in clinical settings. This paradigm of the measurement being anchored to the clinical setting and requiring persons to proactively visit a medical professional to determine their hypertensive status is costly—due to the time and money spent [1]—and considered ineffective—due to the infrequency and error (ie, white coat hypertension) of office BP measurements [2,3]. Hence, we observed remarkable disparities in hypertension detection, treatment, and control across socioeconomic status and race, with populations lacking access to regular office visits and care, having nearly half the awareness of their existing hypertensive status, and enduring up to triple the rates of subsequent cardiac events [4,5]. Technologies that enable frequent, reliable, and accurate measurements of BP in ambulatory settings promise to reduce the global burden of hypertension and offer an opportunity to advance health equity [6]. Leveraging the ubiquity of smartphones and digital health technologies equipped with highly sensitive, miniaturized sensors is essential for the remote monitoring of BP [7].

Existing wearable devices that incorporate noninvasive BP methodologies offer an affordable and efficient means of tracking out-of-office BP [8]. Unfortunately, they commonly use uncomfortable techniques (ie, oscillometry and tonometry) that demand imparting forces on blood vessels to achieve accurate measurements [9-11]. These inconveniences fail to empower users to take control of their health, posing a significant challenge to the widespread routine monitoring of BP. Instead, strategies that compute the pulse transit time (PTT), a measure of arterial stiffness, present a convenient alternative for BP estimation [12].

The PTT, the time the pressure wave propagates along the length of the arterial tree, is a cuffless surrogate for BP and can be acquired noninvasively [12]. In practice, the acquisition of noninvasive PTT requires a combination of sensors—typically an accelerometer, force sensor, light-emitting diode (LED) and photodiode, electrode, or ultrasonic transceiver—placed proximally and distally along the arterial tree and computed from fiducial points in the captured seismocardiogram (SCG), ballistocardiogram, photoplethysmogram (PPG), impedance cardiogram, impedance plethysmogram, or arterial blood pressure (ABP) waveform [13,14]. Despite their inherent convenience, these sensing modalities are naturally of concern when used in populations with intrinsic mechanical, optical, and electrical barriers, stemming from higher melanin levels and body fat percentages.

To the best of our knowledge, noninvasive PTT-based BP estimation has yet to be examined in a diverse population—a gap in our scientific understanding that presents a formidable obstacle to its adoption. Specifically, some medically underserved areas (MUAs), which stand to benefit the most from remote monitoring [15], have a large number of Black and Latino individuals with higher melanin content and obesity rates compared with White individuals [16]. Recent notable data from the Centers for Disease Control and Prevention further stress these concerns by exposing that non-Hispanic Black individuals not only have significantly *higher hypertension prevalence* than non-Hispanic White individuals but also witness significantly *lower hypertension control* rates [17,18]. Affordable remote monitoring options have the responsibility to combat not only social determinants of health, such as access to health care and income, but also in turn the existing health disparities that are byproducts of them. As a result, there exists a glaring hole in PTT-based BP monitoring—that this technology has yet to be tested on the population for whom it may be the most valuable, and until now, its continuing practice will only exacerbate existing health disparities.

### Objectives

In our previous work, we designed a wearable, multimodal, wrist-worn PTT monitoring device (SeismoWatch) and validated it in both controlled lab [19] and unsupervised home [20] settings, primarily on young, healthy persons with lighter skin. In this paper, we expand upon our previous work with a community-engaged research strategy that leverages expertise from a nonprofit organization serving MUAs in Georgia and evaluated our device in a more diverse population. We present our device’s ability to accurately estimate BP in this diverse population and capture significant demographic-level differences in underlying arterial stiffness that coincide with observations from existing literature, through the calibration coefficients used in our BP estimation model. This work represents the first time that a noninvasive, cuffless, PTT-based wearable device has been extensively evaluated in a community-based diverse population as a potentially reliable and convenient monitoring option toward, ultimately, the remote screening and management of hypertension for health equity.

## Methods

### Study Protocol

A comprehensive breakdown of the demographics of the study population is presented in Table 1. This study was conducted under a protocol approved by the Georgia Institute of Technology institutional review board (protocol number H19251). The study was separated into two different populations (N=44 participants) referred to throughout this work as follows: (1) a young and healthy homogeneous population (first cohort=26 participants) and (2) an older, entirely Black, higher BMI, metropolitan population (second cohort=18 participants) recruited later through the help of our community outreach partners—a nonprofit organization serving medically underserved persons in the state of Georgia. For the first cohort, 26 (19 males and 7 females) young and healthy volunteers (mean age 26.7 years, SD 3.7; mean weight 73.8 kg, SD 14.1; height 173.9 cm, SD 9.6; and mean BMI 24.2 kg/m^2^, SD 3.2) with no previous history of cardiovascular disease were recruited, and written informed consent was obtained. For the second cohort, 18 (6 males and 12 females) Black participants (mean age 44.1 years, SD 11.7; mean weight 94.4 kg, SD 18.0; mean height 169.6 cm, SD 11.5; and mean BMI 33.2 kg/m^2^, SD 7.6) with no previous history of cardiovascular disease other than hypertension were recruited from the Atlanta metropolitan area, written informed consent was obtained, and further demographic information was collected post hoc with verbal consent. Both hypertensive status and the use of regular prescription medications were self-reported.

**Table 1:** Participant demographics and cardiovascular parameters for study participants (grouped by cohort; N=44).

Demographics and cardiovascular parameters^a^			Homogenous data set (first cohort; n=26; participant 1-26)		Community outreach (metropolitan Atlanta) data set (second cohort; n=18; participant 27-44)		*P* value	
Age (years), mean (SD)			26.7 (3.7)		44.1 (11.7)		<.001	
**Sex, n (%)**								
	Male	19 (73)		6 (33)		N/A^b^		
	Female	7 (26)		12 (67)		N/A		
Height (cm), mean (SD)			173.9 (9.6)		169.6 (11.5)		.19	
Weight (kg), mean (SD)			73.8 (14.1)		94.4 (18.0)		<.001	
BMI^c^ (kg/m^2^), mean (SD)			24.2 (3.2)		33.2 (7.6)		<.001	
**Obesity class, n (%)**								N/A
	I	1^d^ (4)		2^e^ (11)				
	II	N/A		3^f^ (17)				
	III	N/A		4^g^ (22)				
**Race, n (%)**								N/A
	Black	1^h^ (4)		18 (100)				
	Other race	25 (96)		N/A				
**Hypertensive status, n (%)**								N/A
	Normotensive	26 (100)		15 (83)				
	Hypertensive	N/A		2^i^ (11)				
	Hypotensive	N/A		1^j^ (6)				
**Current medications, n (%)**								N/A
	Hydrochlorothiazide (1×day)	N/A		2^k^ (11)				
	Lisinopril (1×day)	N/A		1^l^ (6)				
	Iron supplement	N/A		1^m^ (6)				

^a^Statistical significance between groups in values, where applicable, was computed using an unpaired two-tailed t test.

^b^N/A: not applicable.

^c^Obesity classified using the BMI per the guidelines from the National Heart, Lung, and Blood Institute of the National Institutes of Health [21] (I: BMI=30-34.9; II: BMI=35-39.9; III: BMI ≥40).

^d^Participant 23.

^e^Participants 30 and 43.

^f^Participants 38, 40, and 42.

^g^Participants 34, 36, 37, and 41.

^h^Participant 5.

^i^Participants 29 and 37.

^j^Participant 33.

^k^Participants 29 and 37.

^l^Participant 29.

^m^Participant 33.

**Figure 1:**
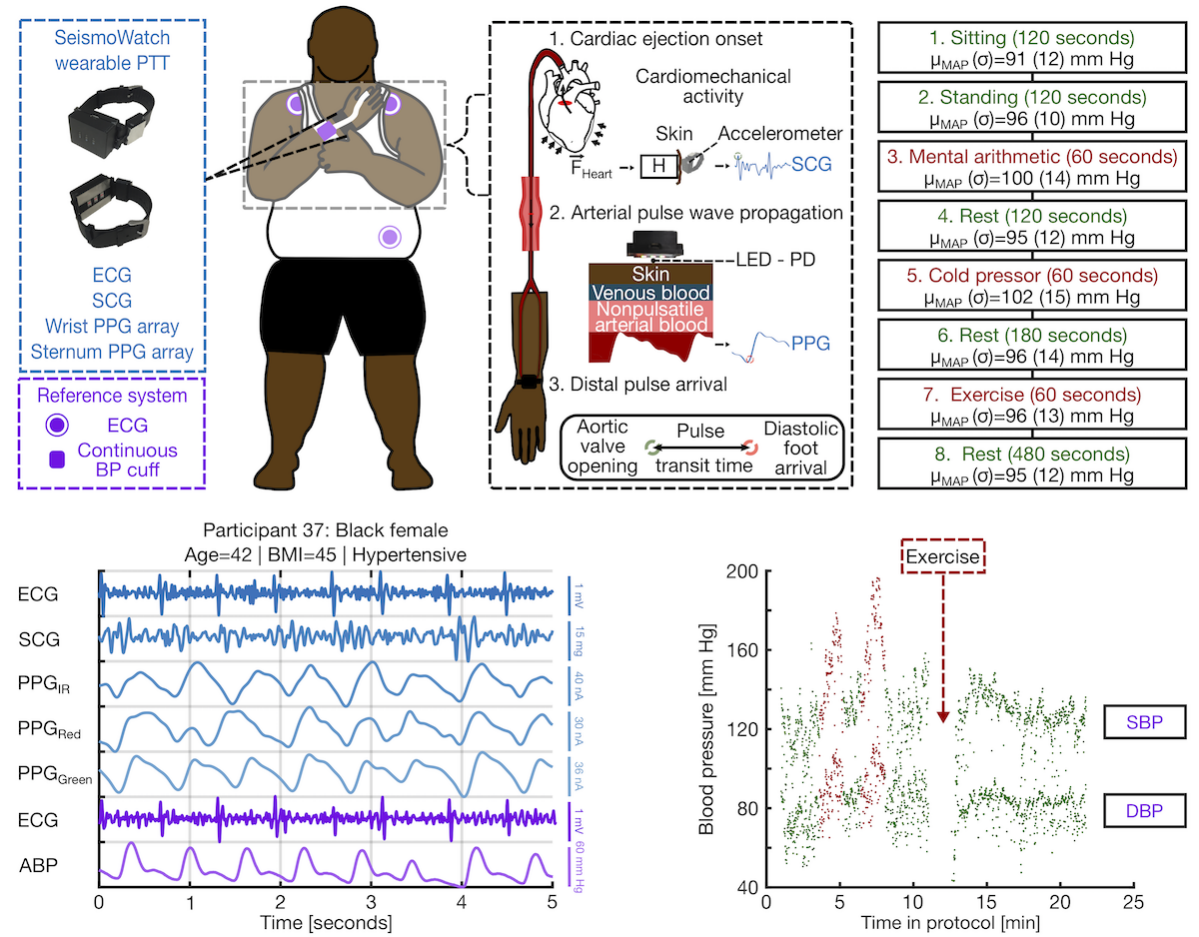
Concept overview and study design. Sensor information and placement locations for wearable system (blue) and reference system (purple). Noninvasive pulse transit time (PTT) measurement concept overview using seismocardiogram (SCG) and photoplethysmogram (PPG) sensors. Study protocol tasks in chronological order with duration and mean (SD) of mean arterial pressure (MAP) values for each task. Sample filtered signals from the participant with the lowest signal-to-noise ratio (SNR) signals (n=37): a hypertensive, high BMI, older Black female. In order from top to bottom: electrocardiogram (ECG), SCG, infrared PPG, red PPG, green PPG signals measured from the wearable system (blue) and the synchronized ECG, and arterial blood pressure (ABP) signals measured by the reference system (purple). Systolic blood pressure (SBP; top) and diastolic blood pressure (DBP; bottom) plotted across the full protocol for participant 37, with rest periods (green) and perturbations used to modulate BP (red) highlighted in chronological order, and the location where the reference finger-cuff continuous blood pressure (BP) system was paused during the exercise indicated. ABP: arterial blood pressure; BP: blood pressure; DBP: diastolic blood pressure; ECG: electrocardiogram; LED: light-emitting diode; PD: photodiode; PPG: photoplethysmogram; PTT: pulse transit time; SBP: systolic blood pressure; SCG: seismocardiogram.

**Figure 2:**
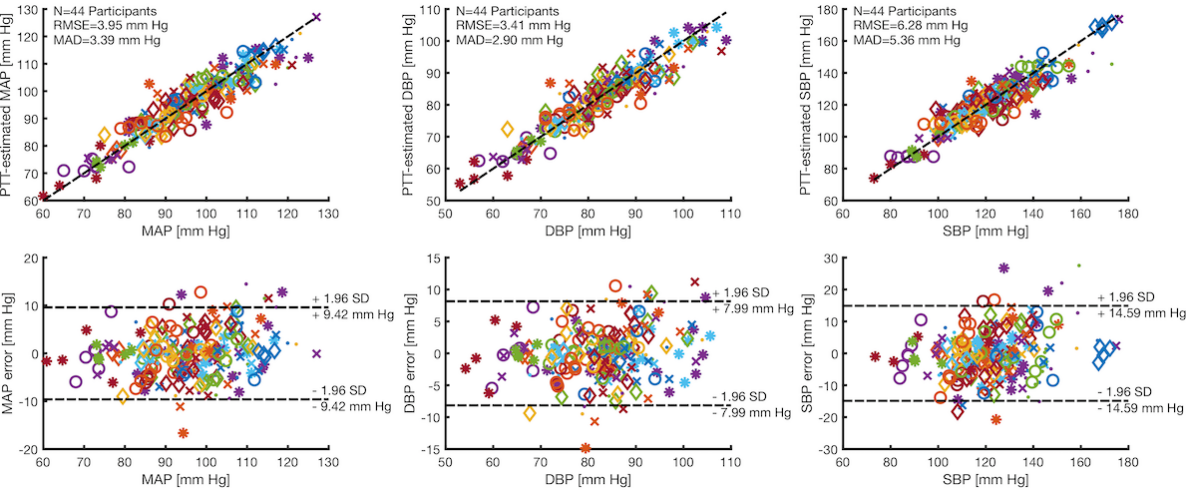
Wearable pulse transit time (PTT)–based blood pressure (BP) estimation results. Correlation and Bland-Altman plots between PTT-estimated BP and the finger-cuff continuous BP for mean arterial pressure, diastolic blood pressure, and systolic blood pressure estimation. The root mean squared error and the mean absolute difference for each correlation are shown. DBP: diastolic blood pressure; MAD: mean absolute difference; MAP: mean arterial pressure; PTT: pulse transit time; RMSE: root mean square error; SBP: systolic blood pressure.

**Figure 3:**
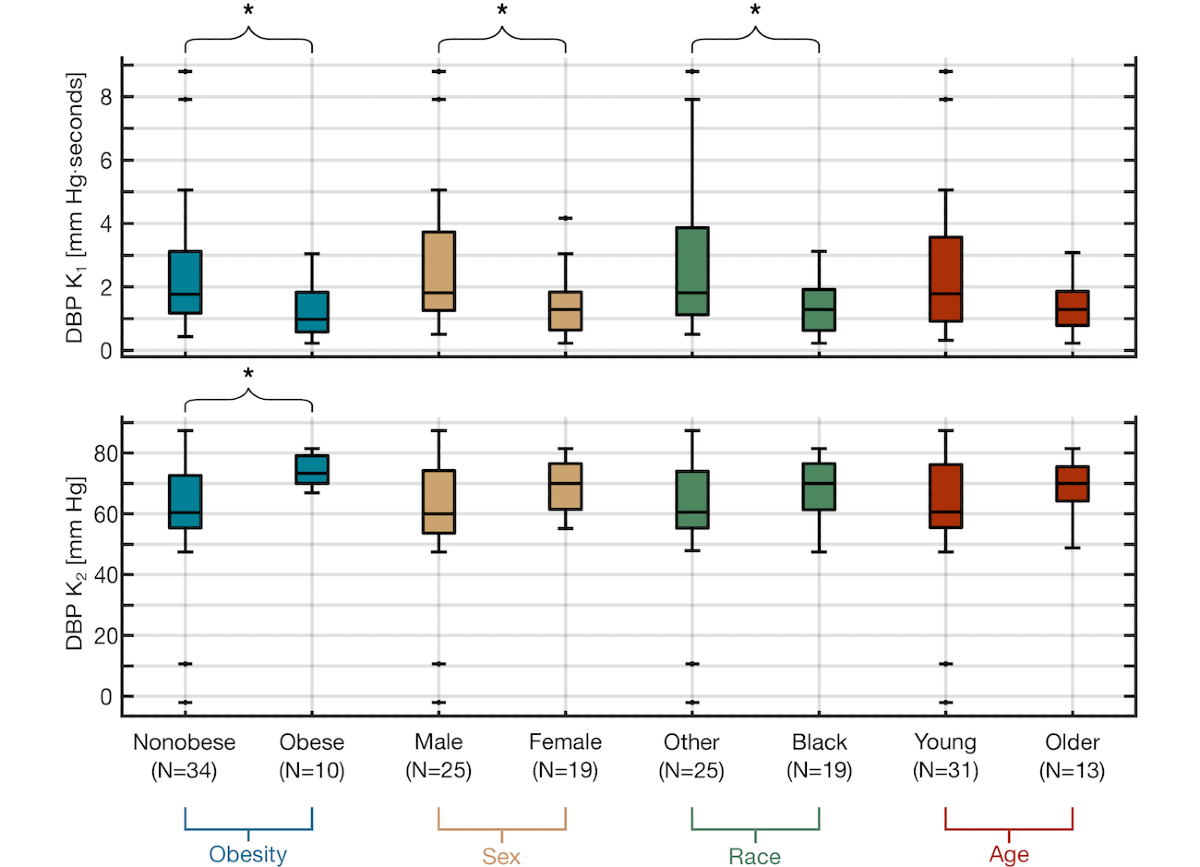
Participant-specific diastolic blood pressure (DBP) calibration coefficients are significantly different in demographics with typical disparities in arterial stiffness. Boxplots showing the statistically significant (*P<.05; Mann-Whitney U) difference in the DBP K1 and K2 calibration coefficients between participants who are nonobese and obese. Boxplots showing the statistically significant (*P<.05; Mann-Whitney U) difference in the DBP K1 calibration coefficients between male and female participants. Boxplots showing the statistically significant (*P<.05; Mann-Whitney U) difference in the DBP K1 calibration coefficients between participants of other race and Black participants. Boxplots showing the difference in the DBP K1 and K2 calibration coefficients between young and older participants. DBP: diastolic blood pressure.

The concept of the study design is shown in Figure 1. Although not explicitly shown, two versions of the SeismoWatch were used in this study: a previous version of the hardware with comparable sensors was used in the young, homogeneous population (ie, the first cohort), before being adapted for a more robust, portable, and multimodal wearable device used in the metropolitan Atlanta population (ie, the second cohort). Specifically, the data from these cohorts were collected during two intervals, between which the hardware was revised to incorporate multiwavelength PPGs before investigating the performance of the sensing modality in the underrepresented population. This was essential to assess the efficacy of shorter-wavelength LEDs (ie, those with shallower skin penetration depths) in a Black population. However, in both the correlations in Figure 2 and calibration coefficient comparisons in Figure 3, only the results derived from the infrared (IR) PPGs, available to both devices, were computed and shown. The other key sensing components and reference system components were essentially identical: (1) the first version of the device used an analog version of the accelerometer (ADXL354, Analog Devices) to acquire the SCG, whereas the second version simply used the digital version of the same sensor (ADXL355, Analog Devices) to reduce size and (2) the finger-cuff continuous BP reference system (ccNexfin, Edwards Lifesciences) along with the data acquisition module (MPU150, Biopac Systems) were identical in both studies.

To acquire a timing reference for the start of a cardiac cycle, while serving as a reference for alignment to the wearable system signals, a wireless electrocardiogram (ECG) module (BN-EL50, Biopac Systems) was attached to the participant in a three-lead configuration with Ag/AgCl gel electrodes as shown in Figure 1. As depicted in Figure 1, a finger-cuff BP sensor based on the volume-clamp methodology (ccNexfin, Edwards Lifesciences) [22,23] was placed on the same hand as the watch, acquiring a reference measurement of continuous beat-by-beat BP. Although volume-clamping continuous BP devices are not the clinical gold standard for ABP measurements, an arterial line was not feasible due to invasiveness, and a sphygmomanometer was not used because of the need for a trained professional and lack of beat-by-beat BP data. Similarly, semiautomated BP cuffs were not used as they hinge on following strict guidelines to obtain an accurate reading, such as being seated and resting the arm at heart level, which were impossible to satisfy simultaneously while acquiring watch measurements, given the need for the contralateral hand to touch the ECG electrode to activate the PTT mode [20]. In addition, it was recently demonstrated that a volume-clamping–based system had comparable accuracy with noninvasive oscillometric BP cuffs [24]. All reference system sensors were sampled at 1 kHz and interfaced to a computer using a data acquisition system (MPU150, Biopac Systems) and its corresponding software (Acqknowledge, Biopac Systems). The reference system files were saved to a desktop computer for postprocessing.

Participants were asked to change into either a V-cut T-shirt or tank top, if not wearing one already, to acquire the sternal PPGs included in the wearable designs, though not examined in this work. The watch was fitted such that the PPGs faced the radial artery on the ventral side of the wrist. To capture the PTT, the participant performed a simple maneuver to place the watch on the sternum to acquire the SCG for the proximal timing reference, as shown in Figure 1, whereas the PPGs were sampled at both the sternum and wrist. Although this offers a noncontinuous assessment, routine remote BP monitoring using oscillometric devices has already demonstrated clinical value, although it does not offer continuous BP measurement [2]. Specifically, ambulatory BP monitors, due to their superior portability and measurement frequency—comparable with what this wearable device can easily provide [20]—have become invaluable for the screening and management of hypertension [2] such that the added benefit of continuous BP measurement may only be marginal.

In order, the participants went through a 2-minute baseline period while sitting before obtaining another 2-minute baseline measurement while standing. Then, a series of perturbations with varying rest periods in between were used to modulate BP. First, a mental arithmetic exercise was used to increase BP [12], in which participants were given a three-digit integer and were told to add the sum of the digits to the number repeatedly for 1 minute. Then, a cold pressor test was conducted in which participants submerged their hand contralateral to the watch in a bucket of ice water for as long as tolerable or until the full minute. Finally, during the exercise session, the finger cuff was removed to avoid damage, and the participant performed either a stair stepping or bicycling exercise, based on personal preference, for 1 minute. As mentioned in our previous work [20], the new version of the watch enters the PTT measurement mode when the user places a finger from their hand contralateral to the watch on the positive wrist ECG electrode; therefore, we were unable to acquire PTT data during exercise for both cohorts and cold pressor for the participants in the second cohort (ie, second cohort). Although with the newer hardware, we were unable to collect PTT data during the cold pressor perturbation for the second cohort, the effect of the cold pressor—assessed directly after the hand was removed from the ice water (ie, a maximum of 1 minute after immersion)—was still well within its physiological window during the following rest period [25]. Overall, as our device is not designed to offer continuous measurements of BP, examining the effect of these perturbations in the rest period directly following them, similar to our previous work [19], still allowed for a comprehensive evaluation of the methodology in a diverse population. However, PTT data from the first cohort during the cold pressor were still used. As the BP data from the cold pressor test were still acquired for the second cohort, as the continuous BP cuff was still on, the mean arterial pressure (MAP) values were factored into the ones displayed in Figure 1. To do so, a 50 ms moving average filter was applied to the measured continuous BP signal, ensemble averages of 10 heartbeats with 50% overlap were taken, and the BP beat with the highest signal-to-noise ratio (SNR) was selected.

### Signal Processing

**Figure 4:**
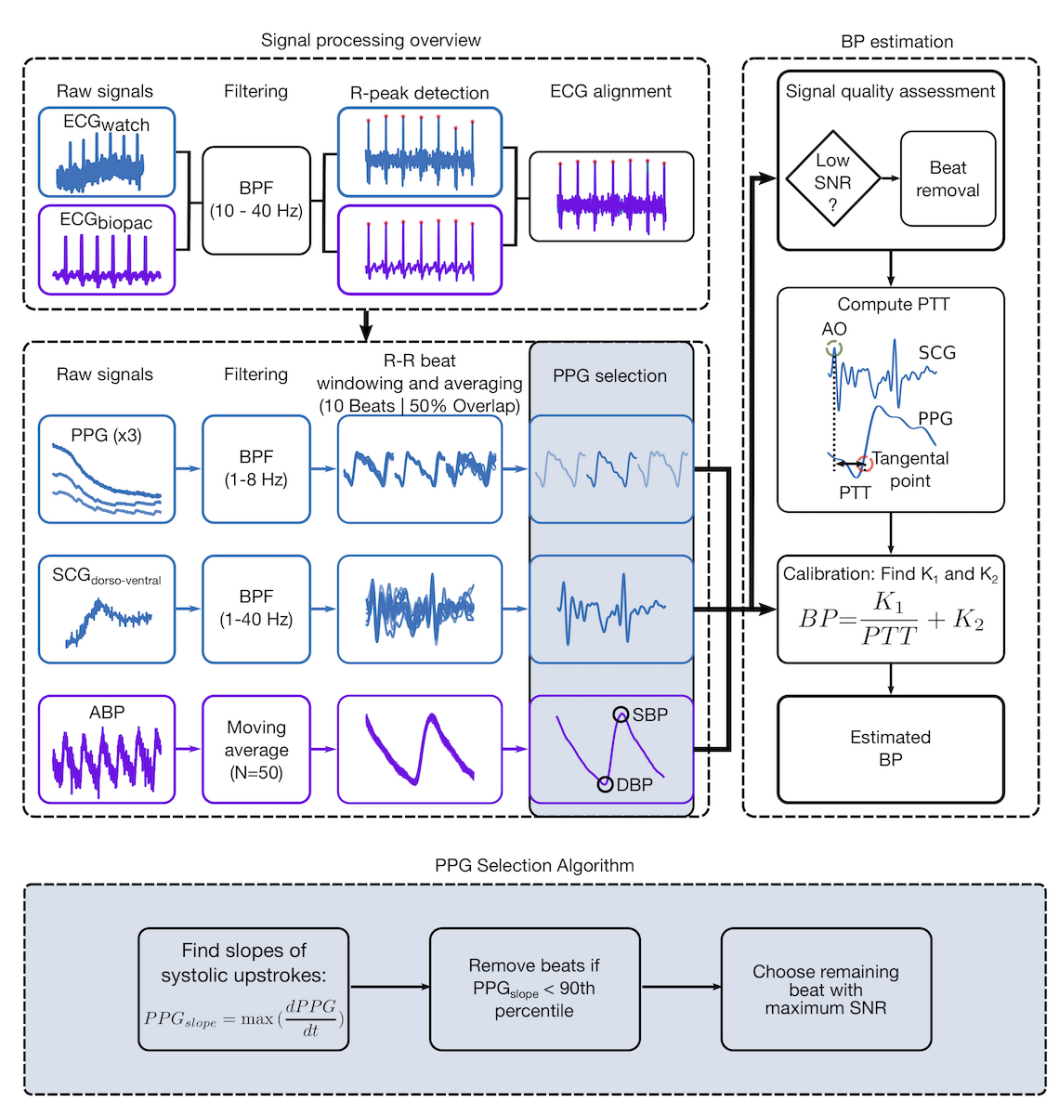
Signal processing pipeline. Block diagram of signal processing overview showing signal alignment using electrocardiogram signals acquired from the wearable system (blue) and reference system (purple) before bandpass filtering, heartbeat windowing, and photoplethysmogram (PPG) selection. After beat selection and signal quality assessment, the pulse transit time is computed as the aortic valve opening point of the seismocardiogram to the diastolic foot of the PPG. Calibration is used to estimate blood pressure (BP) using the arterial blood pressure waveform acquired from the continuous BP finger-cuff. Block diagram of the custom PPG selection algorithm, locating beats with greater systolic upstrokes and signal-to-noise ratio (SNR). ABP: arterial blood pressure; AO: aortic valve opening; BP: blood pressure; BPF: bandpass filter; ECG: electrocardiogram; PPG: photoplethysmogram; PTT: pulse transit time; SBP: systolic blood pressure; SCG: seismocardiogram; SNR: signal-to-noise ratio.

The signal processing pipeline is shown in Figure 4. All signal processing and statistical analyses were performed in MATLAB R2018a (MathWorks). Before preprocessing the SCG and PPG signals acquired from the wearable system, it was imperative to time-align them to the continuous BP signal from the reference system using the respective ECGs to ensure proper temporal comparison. Specifically, the ECGs from each system were first filtered using a digital finite impulse response bandpass filter (BPF; fpass=10-40 Hz) to remove baseline wander due to postural sway and extract the R-wave, which was then identified using a simple peak detection algorithm. Then, cross-correlation was used to align the R-peaks of the two ECG readings by detecting the amount of lead and truncating either the wearable or reference signals depending on the condition. After alignment, the dorsoventral axis of the SCG (ie, z-axis acceleration) and green, red, and IR wrist PPGs were filtered using a digital finite impulse response BPF with bandwidths of 1-40 Hz and 1-8 Hz, respectively, to remove their out-of-band noise and baseline wander due to respiration. In addition, the continuous ABP waveform was smoothed using a 50 ms moving average filter.

Next, the filtered and aligned SCG, PPG, and ABP waveforms were split into separate heartbeats using the detected R-R intervals of the synchronized ECG. Then, these heartbeat-indexed signals were ensemble-averaged using 10-beat windows with 50% overlap before assessing the signal quality to select the highest quality beat per task for each participant, similar to the methods used in our previous works [19,20]. Given the number of high BMI participants in this population, the SCG not only had a lower mean SNR when compared with our previous studies but was also observed to have less variability than the PPG SNR; hence, an emphasis was placed on determining the optimal PPGs first. In addition, upon an initial assessment of signal quality, it was observed that when the PPG signal had the highest SNR, typically, the SCG signal did as well—perhaps because acquiring a clean PPG signal inherently hinges on applying consistent pressure. The optimum PPG was selected using a physiologically inspired algorithm to first identify the beats with the top 10% of systolic upstrokes (ie, maximum of the derivative of the PPG waveform) and then select the remaining beat with the maximum SNR. The SNR was calculated using a noise-to-signal ratio detection algorithm detailed in Inan et al [26]. The methods used to determine the timing references for PTT calculation, the foot of the PPG, and the aortic valve opening (AO) point of the SCG were the same as those used in our previous studies [19,20]. Specifically, the foot of the PPG was computed using the intersecting tangent method described in the study by Mukkamala et al [12], and the AO point was assumed to be the first peak in each ensemble-averaged window before the foot of the PPG. Occasionally, the SCG signal was manually annotated to impose realistic constraints for the AO point or to ensure that the same morphological peak was consistently chosen for all tasks per participant. Participant-specific SNR thresholds were set to retain only high-fidelity signals; if the SNR of the SCG, PPG, or ABP beats was not greater than the prescribed cutoff, or if the foot of the PPG was not within a realistic range, then the respective ensemble-averaged waveforms were deemed too noisy for use and that task was not used for PTT calculation. Notably, the continuous reference BP allowed for the ability to evaluate the SNR of the ABP signal and incorporate this quality assessment into our signal processing pipeline to remove beats with low SNR reference measurements. After the entire signal quality assessment process, at least four of the tasks were used for BP estimation per participant. Finally, the PTT was calculated as the difference of the proximal timing reference, AO point of the paired SCG, and distal timing reference, the foot of the selected PPG. In addition to wavelength comparisons, the green and red wavelength PPGs were not used as the IR wavelength wrist PPGs had the highest mean SNR, because of the greater indifference of the IR wavelength to melanin absorption and the ability to capture more pulsatile arteries deeper in the tissue than cutaneous capillaries [12,27].

In addition, the postexercise recovery period was separated into an early and late rest period based on when the BP reached a consistent value. This allowed us to capture both the immediately heightened cardiac output–induced BP increase postexercise and the recovery back to baseline, while opportunely adding another PTT and BP data point for linear regression.

### Statistical Analysis

Simple linear regression was performed independently between wearable participant-specific inverse PTT (PTT^−1^) and reference diastolic blood pressure (DBP), MAP, or systolic blood pressure (SBP) value pairs, to calculate the calibration coefficients necessary to estimate each of the three BP components per participant; nonlinear models, whereas potentially more accurate, dictate the need for more calibration points [12,28]. Therefore, the resulting calibration coefficients—used to estimate BP from the conventional PTT-based BP estimation model shown in equation 1—are merely the slope (ie, slope calibration coefficient [K_1_]) and y-intercept (ie, Y-intercept calibration coefficient [K_2_]) of the line of best fit [12]. This was identical to the calibration methods used in our previous work [19,20].

*BP* = (*K*_1_ / *PTT*) + *K*_2_** (1)**

The mean absolute difference (MAD) was computed from the mean of the absolute value of the difference between the estimated and reference BP. The benchmarks for MAD were chosen based on the Institute for Electronics and Electrical Engineers (IEEE) standard for wearable cuffless BP measuring devices [29]. In addition, the root mean square error (RMSE), calculated as the root mean square of the difference between the estimated BP and measured BP, was computed because of its enhanced sensitivity to outliers.

We stratified the entire study population for the demographic comparisons of the calibration coefficients shown in Figure 3, based on four factors (ie, obesity, sex, race, and age) known to affect arterial stiffness [30-35] and therefore the PTT. The participants were split into nonobese and obese groups based on the guidelines from the National Heart, Lung, and Blood Institute of the National Institutes of Health defining a BMI ≥30 kg/m^2^ as obese [21]. Thus, the nonobese group had a BMI ≤30. To assess differences due to age, we separated the participants into younger (aged ≤40 years) and older groups (aged ≥40 years). Statistical significance (*P*<.05) between demographic data for each cohort was assessed using an unpaired two-sample, two-tailed *t* test, as shown in Table 1.

For the demographic DBP calibration coefficient comparisons, a one-sample Kolmogorov-Smirnov test was used on each data point to test for normality, which determined that none of the data for the comparisons were normally distributed. Then, a Mann-Whitney U test (ie, Wilcoxon Rank Sum test) was used to assess statistical significance (*P*<.05) among the unpaired data.

For the PPG wavelength DBP estimation comparisons—only applicable to the second cohort population due to the differences in hardware used—first, a one-sample Kolmogorov-Smirnov test was used on each data point to test for normality, which determined that none of the data for the comparisons were normally distributed. Then, a Wilcoxon Signed Rank test was used to assess statistical significance (*P*<.05) among the paired data.

## Results

### Multimodal Engineering Mechanics of the SeismoWatch

The previous version of the watch, not shown in this work, consisted of a 3D printed case embedded with an accelerometer, PDs, and IR LEDs. All sensors were connected to a small external circuit box with straps for the participant to wear around the waist. The output of the analog accelerometer (ADXL354, Analog Devices) was connected to an analog front end (AFE) in the circuit box. To amplify the SCG signal and prevent saturation of the alternating current components owing to the varying direct current levels, the AFE separated the direct current and alternating current components using a low pass (fc=1 Hz; G=−10 dB) and BPF (fpass=0.2 Hz-40 Hz) in parallel. An analog adder recombined both components. For PPG measurements, the cathode of the PDs (S2386-18k, Hamamatsu Photonics) was connected to transimpedance amplifiers configured as a low-pass filter (fc=12 Hz; G=110 dB) followed by gain and filter stages (fpass=0.5-12 Hz; G=59 dB). Finally, the ECG was acquired by placing 3 copper dry electrodes on the wrist band of the watch with 2 on the inside in contact with the wrist and 1 on the outside to place the index and middle finger. The 2 on the inside act as the right leg drive electrode and the positive lead, whereas the outside electrode is the negative lead. All electrodes were connected to an AFE (AD8232, Analog Devices) for ECG measurements. A microcontroller (Teensy 3.6, PJRC LLC) sampled the output of the accelerometer, PPG, and ECG AFE at 1 kHz. An onboard SD card was used to store the raw data for postprocessing, and a 1.2 Ah lithium-ion rechargeable battery was used to power the system. All instrumentation details were adopted from our previous work, with minor revisions [19].

**Figure 5:**
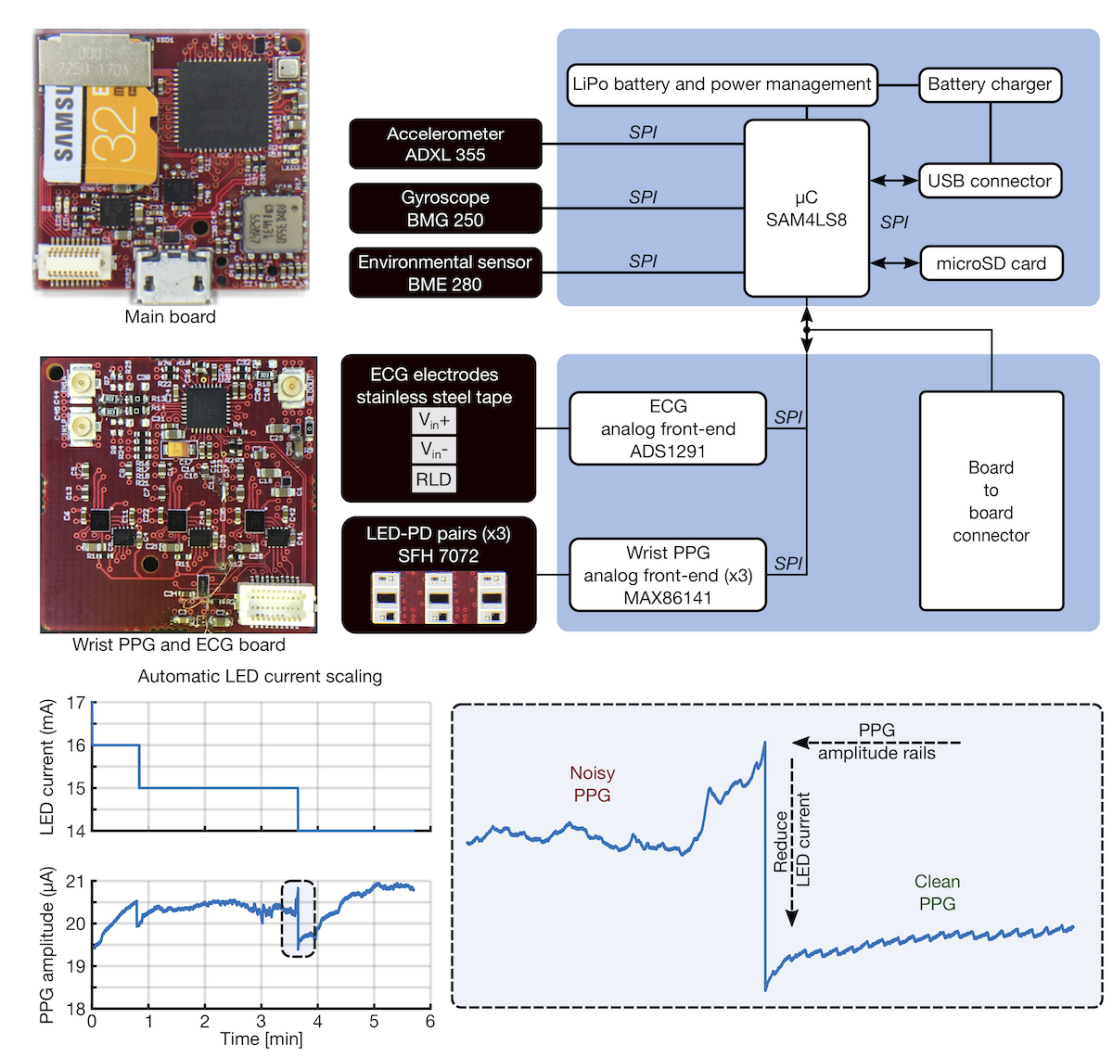
Pertinent multimodal hardware block diagram and adaptive light-emitting diode (LED) scaling. Main board with ATSAM4LS8 microcontroller (µC), ADXL355 triaxial accelerometer, BMG250 triaxial gyroscope, and BME280 environmental sensor using the serial peripheral interface for fast communication supporting higher sample rates. Sensor board used to acquire wrist photoplethysmogram (PPG) and electrocardiogram signals. Automatic LED current scaling in operation during data collection: showing an increase in contact pressure and subsequent saturation of the photodiode, mitigated by an automatic decrease in LED current and overall consequential improvement in PPG signal quality. ECG: electrocardiogram; LED: light-emitting diode; PD: photodiode; PPG: photoplethysmogram; RLD: right leg drive; SD: Secure Digital; SPI: Serial Peripheral Interface.

The updated hardware, pictured in Figure 5, added modalities of sensing (ie, a gyroscope), included multiple wavelengths of LEDs for comparison with IR, improved the form factor for comfort and ease of use, and featured embedded systems innovations leveraged in this study. A more thorough description of the revised hardware is available in our most recent work [20]. An example of the serviceable automatic LED current scaling algorithm, detailed in our previous work [20], is highlighted in Figure 5. This proved to be an integral part of enabling this work; by adaptively adjusting the LED drive current, we were able to prevent saturation and variable PPG signal quality caused by varying contact pressure and, more importantly, prominent differences in skin tone among participants.

### Human Subject Studies in a Diverse Population

All applicable results are presented as mean (SD). Figure 2 illustrates the correlation and Bland-Altman plots for our wearable PTT-based BP estimation of MAP, DBP, and SBP across all participants (N=44). The MAD was 2.90 mm Hg, 3.39 mm Hg, and 5.36 mm Hg for DBP, MAP, and SBP, respectively. The mean RMSE was 3.41 (SD 2.01) mm Hg, 3.95 (SD 2.42) mm Hg, and 6.28 (SD 3.44) mm Hg for DBP, MAP, and SBP, respectively. DBP and MAP estimation had better 95% CIs than SBP at 7.99 mm Hg, 9.42 mm Hg, and 14.59 mm Hg, respectively. The mean Pearson correlation coefficient (PCC) was 0.67 (SD 0.16), 0.63 (SD 0.31), and 0.50 (SD 0.41) for PTT-based DBP, MAP, and SBP estimation, respectively.

The MAD for the individual study populations (first cohort=26 participants and second cohort=18 participants) was 2.69 mm Hg and 3.20 mm Hg, 3.21 mm Hg and 3.64 mm Hg, and 5.17 mm Hg and 5.63 mm Hg, for DBP, MAP, and SBP estimation, respectively. The mean RMSE for the individual study populations (first cohort=26 participants and second cohort=18 participants) was 3.19 (SD 1.64) mm Hg and 3.73 (SD 2.48) mm Hg, 3.78 (SD 2.06) mm Hg and 4.18 (SD 2.90) mmHg, and 6.26 (SD 3.25) mm Hg and 6.32 (SD 3.80) mm Hg for DBP, MAP, and SBP estimation, respectively. The mean PCC for the individual study populations (first cohort=26 participants and second cohort=18 participants) was 0.69 (SD 0.15) and 0.65 (SD 0.17), 0.68 (SD 0.23) and 0.55 (SD 0.38), and 0.58 (SD 0.33) and 0.39 (SD 0.49) for DBP, MAP, and SBP estimation, respectively.

The MAD for the 19 Black participants was 3.18 mm Hg, 3.72 mm Hg, and 5.84 mm Hg for DBP, MAP, and SBP estimation, respectively. The mean RMSE for all 19 Black participants was 3.72 (SD 2.41) mm Hg, 4.29 (SD 2.86) mm Hg, and 6.69 (SD 4.03) mm Hg for DBP, MAP, and SBP estimation, respectively. The mean PCC for all 19 Black participants was 0.64 (SD 0.17), 0.53 (SD 0.38), and 0.37 (SD 0.48) for DBP, MAP, and SBP estimation, respectively.

The MAD for the 10 participants who were obese was 2.69 mmHg, 3.17 mm Hg, and 5.02 mm Hg for DBP, MAP, and SBP estimation, respectively. The mean RMSE for all 10 participants who were obese was 3.28 (SD 2.59) mm Hg, 3.69 (SD 3.00) mm Hg, and 5.71 (SD 4.18) mm Hg for DBP, MAP, and SBP estimation, respectively. The mean PCC for all 10 participants who were obese was 0.65 (SD 0.18), 0.52 (SD 0.48), and 0.39 (SD 0.58) for DBP, MAP, and SBP estimation, respectively.

Figure 3 depicts the boxplots of the DBP calibration coefficients from our estimation model, K_1_ and K_2_, for four different demographic factors known to affect arterial stiffness: obesity, sex, race, and age. The DBP K_1_ and K_2_ values for nonobese (N=34) versus obese (N=10) participants are 2.38 (SD 1.99) mm Hg/s versus 1.20 (SD 0.88) mm Hg/s and 61.02 (SD 18.03) mm Hg versus 74.31 (SD 5.14) mm Hg, respectively. The DBP K_1_ and K_2_ values for male (N=25) versus female (N=19) participants are 2.65 (SD 2.18) mm Hg/s versus 1.40 (SD 0.98) mm Hg/s and 60.16 (SD 20.64) mm Hg versus 69.14 (SD 8.29) mm Hg, respectively. The DBP K_1_ and K_2_ values for non-Black (N=25) versus Black (N=19) participants are 2.63 (SD 2.21) mm Hg/s versus 1.44 (SD 0.94) mm Hg/s and 60.66 (SD 20.29) mm Hg versus 68.49 (SD 9.98) mm Hg, respectively. The DBP K_1_ and K_2_ values for young (N=31) versus older (N=13) participants are 2.38 (SD 2.09) mm Hg/s versus 1.47 (SD 0.87) mm Hg/s and 61.96 (SD 19.03) mm Hg versus 69.00 (SD 9.22) mm Hg, respectively.

Both K_1_ and K_2_ were significantly different between the nonobese and obese populations (*P*=.045 and *P*=.008, respectively). The female K_1_ values were significantly (*P*=.04) lower than those of their male counterparts. The K_1_ values for Black participants were significantly (*P*=.047) lower than those of the other races.

For the participants in the second cohort—all Black—with whom we used the newer version of the hardware [20] that included green and red LEDs in addition to the IR, the PCC for DBP estimation was 0.38 (SD 0.34), 0.59 (SD 0.44), and 0.65 (SD 0.17) when using the green λ=526 nm, red λ=660 nm, and IR λ=950 nm wavelength PPGs for the distal timing reference, respectively. The PCC for the IR and red wavelength PPGs was significantly (*P*=.01 and *P*=.048) higher than that of the green wavelength PPGs. However, the corresponding mean DBP RMSE was 3.95 (SD 2.53) mm Hg, 3.11 (SD 2.33) mm Hg, and 3.73 (SD 2.48) mm Hg for green, red, and IR, respectively.

## Discussion

### Principal Findings

To the best of our knowledge, this is the first study to accurately estimate DBP and MAP using noninvasive PTT measurements acquired from a holistic population, with considerable differences in body fat percentage, melanin levels, and vascular stiffness associated with age and hypertension. Furthermore, our SBP estimation is sufficient to be clinically recommended for monitoring [29,36]. We demonstrated the reliability of a convenient method for estimating BP and observed that our calibration coefficients were significantly different in characteristic demographic groups known to have increased arterial stiffness. This work represents a necessary advancement toward remote monitoring for persons in MUAs by enabling wearable PTT-based BP estimation, including through the comprehensive evaluation of a watch-based form factor conducive to obtaining ambulatory BP measurements in low-resource settings.

### Accurately Estimating BP in a Diverse Population Using a Multimodal Wearable Device

We demonstrated the performance of our wrist-worn PTT-based device when used to estimate BP within a diverse population over the course of multiple unique perturbations. Our results for MAP and DBP passed the acceptable benchmarks for the BP estimation error set by the IEEE standard on wearable cuffless BP estimation devices (MAD≤5 mm Hg) [29]. We were still able to achieve a reliable correlation between PTT and BP even with several demographic factors such as age, melanin levels, and BMI inherently influencing the measured optical-PPG and mechanical-SCG signals.

The DBP estimation remained the most accurate, similar to our previous studies [19,20]; the foot of the PPG waveform, used as the distal timing reference, indicates the arrival of the pulse wave during end *diastole*. Similarly, the SBP estimation continued to perform the worst, as the peak of the pulse wave is the fiducial marker of the PPG that occurs during *systole*; however, the peak is not frequently extracted, as its true timing can be confounded by wave reflection interference, leading to unreliable PTT estimates [12]. Recent studies have demonstrated that the PTT computed using the diastolic foot of the PPG outperforms that using the systolic maximum for both DBP and SBP [37].

The DBP RMSE was relatively similar at both low and high values of DBP, which indicates that the diastolic foot was a dependable timing reference for calculating the PTT, irrespective of inherent participant-specific differences in BP. Although our SBP estimation was just outside the acceptable limits set forth by the IEEE standard (ie, MAD=5.36 mm Hg vs 5.0 mm Hg) [29], this error translates to a grade B classification [29] and therefore would still be clinically recommended for monitoring SBP [36]. Furthermore, the SBP range studied in this work was greater than 100 mm Hg, substantially higher than that reported in previous studies in the literature for wearable cuffless BP estimation, and a combination of different perturbations was used to modulate BP. Using a single perturbation would have led to an improved correlation [12,14], as in our previous work where we had only used exercise [19]. However, a comprehensive evaluation of this methodology would be incomplete without a procedure consisting of a wide variety of perturbations with different known physiological responses and pathways to modulate BP [14]. In addition, as noted in Figure 1, the exercise perturbation did not apparently produce a marked difference in BP due to several factors: (1) technical limitations in rapid calibration for the reference measurement (ie, finger-cuff continuous BP) and increased motion artifacts following exercise led to a greater percentage of beat removal in the early exercise section than any other task and (2) exercise does not necessarily consistently modulate BP in a predictable manner due to differences in participant-specific vasoactivity and contractility [12,38].

Only the DBP was examined for further analyses conducted below because, as previously mentioned, the distal timing reference used in this work (ie, the foot of the PPG waveform) occurs during diastole and therefore provides the most reliable estimation of DBP out of the three BP components [12]. The dependability of the diastolic foot and our robust DBP estimation were necessary before performing in-depth analyses with confidence. Although elevated SBP is considered to be the greatest predictor of future cardiovascular risk [39,40], elevated DBP has nonetheless been shown to independently increase the risk of subsequent cardiac events [39,41]. In addition, DBP is a greater contributor to MAP, which in older patients with isolated systolic hypertension, when compared with an equivalent increase in pulse pressure, has been shown to be a comparable independent predictor of both stroke and all-cause mortality [42]. Finally, DBP has been shown to be a more significant predictor than SBP of new-onset hypertension in adults aged ≤50 years [40,43-45]. This suggests that accurate DBP estimation using a wearable device can efficiently be used to incentivize people to make healthy lifestyle modifications *earlier in life*, central to the World Health Organization’s effort to reduce the global prevalence of hypertension [46].

### Essential Device Novelties Enabling Reliable PTT Computation

For the first time, we demonstrated that noninvasive PTT measurements are reliable estimators of BP across a wide range of skin tones and BMI. Both DBP and MAP estimation for the 10 participants who are obese and 19 Black participants in this study were well under the IEEE requirement [29]. This was enabled by the highly sensitive hardware, multisensor approach, and automated LED current scaling that our custom wearable device offers [20]. The PPG array and adaptive LED current scaling allowed us to automatically mitigate poor signal quality issues due to misplacement, inherent differences in skin tone, and applied pressure that typically corrupts PPG signals. However, the most integral components of our PPG hardware were the IR wavelength LEDs.

We leveraged longer wavelengths of light for deeper penetration into the tissue to robustly acquire the PPG signal from arteries located deeper than the cutaneous vascular bed [12]. Cutaneous arteries are greatly affected by the changes in vascular tone expected from the perturbations we used to modulate BP herein (ie, cold pressor and exercise). Furthermore, as IR PPGs are more susceptible to motion artifacts than lower wavelength ones [12,47], our PPG-first signal quality assessment not only avoided these motion artifact corrupted waveforms because of their low SNR but also avoided moments where the SCG quality would naturally suffer as well. However, even the red PPGs had a considerably larger SD in their PCC than the IR PPGs, possibly because the IR wavelength, when compared with red, is less sensitive to the oxygen content of hemoglobin and has approximately half the skin absorption coefficient in Black individuals [12,27]. Despite statistically significant differences in the PCC using IR and red PPGs rather than green PPGs, the actual DBP RMSEs were comparable. This implies that when using the green PPGs for participants with a low PCC, our signal quality assessment algorithm removed beats with greater BP variability, resulting in a lower SD of DBP and consequent RMSE. Although even green wavelength PPGs have demonstrated the ability to reliably extract heart rate across a wide variety of skin tones [48], our data suggest that these shorter wavelengths cannot be used to dependably compute the PTT in a diverse population. In addition, although unconventional, our watch was placed on the ventral side of the wrist, which allows for both higher quality, convenient SCG acquisition and enhanced PPG SNR due to viable access to the radial artery and less melanin content than the dorsal side [49]. Therefore, existing smartwatches, beginning to slowly incorporate cuffless, noninvasive BP methodologies, may face even greater difficulties in achieving accurate PPG measurements across a broad range of skin tones.

Finally, our physiologically inspired PPG selection algorithm—to first select the PPG signals with the greatest systolic upstrokes—had an important role in reducing the BP estimation error. PPG waveforms with greater systolic upstrokes (ie, maximum derivative of the PPG waveform) offer improved PTT estimates and are key indicators of BP stemming from larger, more pulsatile, elastic arteries with greater distensibility [12,50]. In addition, several recent machine learning (ML) approaches to use the PPG signal for BP estimation have shown that the systolic upstroke is one of the most important features of the waveform [51,52]. Hence, the selection algorithm, by extracting information from these more reliable and clinically important arteries, was a central part of our ability to notice the demographic differences in arterial stiffness rooted in our calibration coefficients.

### Calibration Coefficients Capture Demographic Differences in Arterial Stiffness

We observed that the participant-specific calibration coefficients used in the standard linear PTT-BP estimation model for DBP, shown in equation 1 (ie, K_1_ and K_2_), are significantly different between subpopulations with large variations in demographic factors known to affect arterial stiffness. We selected the four demographic categories (ie, obesity, sex, race, and age) based on the literature, emphasizing these as major determinants of differences in arterial stiffness and therefore risk factors for hypertension [16,30-33,35,53,54].

The K_1_ value (ie, the slope of the line of best fit) is indicative of the underlying baseline vascular stiffness, whereas K_2_ (ie, the intercept) represents the inherently correlated bias in baseline BP [12,55,56]. At the same BP, persons with greater arterial stiffness have inherently faster pulse wave velocities (PWVs) and therefore shorter PTTs than persons with normal arterial stiffness [12]. The K_1_ value mitigates these differences in PTT-based estimation by capturing the intrinsic participant-specific arterial stiffness to output similar B*P* values. Therefore, with increasing arterial stiffness, we expected to find a lower K_1_ value and a higher K_2_ value, as observed in the PWV literature [55,56].

Obesity was the only comparison for which the differences in the K_1_ and K_2_ calibration coefficients were statistically significant. This coincides with the literature stating that obesity is one of the greatest age-normalized risk factors and contributors to arterial stiffness [57]. Otherwise, only the K_1_ values in the sex and race comparisons were statistically significant between the groups. Although it has been shown that both females and Black individuals have greater arterial stiffness than similar-age males and White individuals [31,34,35], these two comparisons should be re-evaluated after increasing our recruitment. Approximately 47% (9/19 participants) of both the female and Black population were also obese. The age comparison was not statistically significant, although the older population followed a similar trend of a lower K_1_ and higher K_2_. This finding is not surprising, as significant differences in arterial stiffness and substantial augmentations in arterial remodeling are typically examined in participants aged ≥50 years [32,58].

### Limitations and Future Work

#### Refining Population Demographics and Investigating PTT, K1, and K2 as Potential Digital Biomarkers of Arterial Stiffness

Overall, although this data set captured a more representative population in the range of end users for which consistent BP monitoring is recommended [59], our PTT-based device should be further tested in an exclusively older (ie, age >50 years), morbidly obese (ie, BMI >40 kg/m^2^), and hypertensive population—with even distributions across sex, race, and skin tones along the Fitzpatrick scale—to truly understand the limits of this technology and supplement the findings herein.

Early vascular remodeling due to the demographic factors investigated in this work, not to mention socioeconomic factors affecting MUAs [15,16], predispose individuals who are obese and Black individuals to greater lifetime cardiovascular risk [30,35,57,60,61]. Therefore, future PTT-based BP estimation studies should closely monitor the calibration coefficients, K_1_ and K_2_, as potential intermediate digital biomarkers for longitudinal monitoring and the comparison of arterial stiffness among different persons [7]**.** Eventually, even PTT measurements, as PWV is already an independent predictor of arterial stiffness [62], may indicate subclinical differences in vascular resistance due to early stage arterial remodeling, the main precursor to hypertension [32].

#### Reducing the Burden of Calibration

Consistent recalibration poses a practical concern for PTT-based BP estimation. Hence, future studies should focus on evaluating the timeframe for which participant-specific calibration curves can reliably estimate BP and whether interparticipant and population-level curves can be sufficient. However, given the value of interpreting the calibration coefficients presented in this work, caution should be exercised due to the trade-off of sacrificing this potential usefulness when using generalized interparticipant models. Furthermore, the individual effects of the perturbations used to modulate BP in this experiment should be scrutinized, along with other exercises shown to substantially change BP [63-65]. The goal is to use perturbations that can consistently be leveraged to increase the dynamic range of BP measurements for calibration—critical to achieving optimal estimations at home in our previous work [20] and are achievable in low-resource settings.

#### Leveraging ML and Hardware Advancements for Robust SCG AO Detection

Similarly, to the instrumental role of the physiologically inspired PPG selection algorithm in this work, further exploration into automated SCG fiducial point detection algorithms may help extract the most informative SCG signals. Specifically, the SCG can be greatly affected by inaccurate placement of the watch; however, recent advancements using ML techniques have shown that the SCG waveform is modulated in a predictable manner during these placement inaccuracies [66]. Therefore, by interpreting these findings, one might be able to convert the measured SCG to the archetypal SCG or use a template-matching localization approach [67] for each participant before extracting salient features from the optimal waveform.

In addition, annotating the exact AO point can be challenging because the signal not only has appreciable interparticipant variability, especially in a population with considerable differences in BMI, but can also be corrupted by motion artifacts. Although our technique for extracting the AO point has led to a high correlation between PTT and BP, in both our recent work [20] and this one, for a few sessions, we manually annotated the SCG to impose realistic constraints for the range of the pre-ejection period (PEP) and selected a consistent morphological peak across all tasks per participant. Eventually, robust identification of this timing reference is necessary for reliable automatic PTT computation, as the main advantage of using the PTT over the pulse arrival time (ie, the time from the R-wave of the ECG to the diastolic foot of the PPG) for BP estimation is its ability to account for changes in the nonnegligible cardio-electromechanical delay, that is, the PEP [12,68]. Furthermore, examining the other sensor data available at our disposal, such as filtering the SCG in a higher bandwidth (ie, fpass=30-125 Hz) to retain the phonocardiogram signal indicative of valve closures, using the three-axis gyrocardiogram or simply the other axes of the SCG, could prove to help with improving PEP estimation as shown in previous work [19,69].

### Conclusions

We have demonstrated that a wrist-worn device, using noninvasive PTT estimates, can reliably and conveniently track BP in a diverse population. Leveraging the ubiquity of wearable devices can empower users to make healthy lifestyle modifications such as exercise, which can contribute to a significant reduction in arterial stiffness [30,70] by providing consistent feedback on progress [71-73]. In addition, digital health technologies that accurately estimate BP could potentially be used to titrate BP medications for patients with hypertension from the comfort of their homes [7,74]. In addition to these broader impacts, the knowledge gained from this study—especially when combined with the advent of low-profile, flexible electronics capable of robustly detecting physiological biosignals [75-78]—represents a significant step toward the unobtrusive monitoring of BP in ambulatory settings and health equity for persons in MUAs.

## Abbreviations

ABP: arterial blood pressure
AFE: analog front end
AO: aortic valve opening
BP: blood pressure
BPF: bandpass filter
DBP: diastolic blood pressure
ECG: electrocardiogram
IEEE: Institute for Electronics and Electrical Engineers
IR: infrared
LED: light-emitting diode
MAD: mean absolute difference
MAP: mean arterial pressure
ML: machine learning
MUA: medically underserved area
PCC: Pearson correlation coefficient
PEP: pre-ejection period
PPG: photoplethysmogram
PTT: pulse transit time
PWV: pulse wave velocity
RMSE: root mean square error
SBP: systolic blood pressure
SCG: seismocardiogram
SNR: signal-to-noise ratio
